# Factors influencing quality of life (QOL) amongst elderly caregivers of people living with HIV/AIDS in Phayao province, Thailand: a cross-sectional study

**DOI:** 10.12688/f1000research.16892.1

**Published:** 2019-01-09

**Authors:** Pitakpong Punta, Ratana Somrongthong, Ramesh Kumar

**Affiliations:** 1 College of Public Health Sciences,, Chulalongkorn University, Bangkok, 10330, Thailand; 2 Department of Public Health, Health Services Academy, Islamabad, 44000, Pakistan

**Keywords:** Influencing factors, Quality of life, Elderly, HIV/AIDS

## Abstract

**Background:** There are many impacts on quality of life among elderly people living with HIV patients. This study aimed to assess factors influencing quality of life among elderly people living with HIV/AIDS in a northern province of Thailand.

**Methods: **This cross-sectional study was conducted in Phayao province, Thailand. A systematic sampling technique was employed to select study participants. 152 elderly participants aged 60 years and older with a family member living with HIV/AIDS were recruited to the study. They were interviewed using the World Health Organization Quality of Life-Older Adults Module (WHOQOL-OLD) questionnaire. Stepwise multiple regression analysis was performed to determine the factors influencing quality of life among elderly people affected by family member living with HIV/AIDS.

**Results: **The results of the study showed the mean age of elderly participants was 67.20 + 52 years, most of which were female (97 persons, 63.8%). The mean time taking care of HIV/AIDS patients was 6.61 + 4.96 years. In term of health status among the elderly participants, the majority did not have chronic diseases (61.4%), amongst those with chronic diseases (38.6%), hypertension and diabetes were the most common. The average quality of life score was at a fair level.  The time taking care of HIV/AIDS patients and health status were significant predictors of quality of life among participants 8.1 % ( *R ^2^*=.081; *p *< .05).

**Conclusion: **In order to improve quality of life among elderly caregivers to family member living with HIV/AIDS, time taking care of HIV/AIDS patients and health status should be focused on, amongst other factors. Help and support from the government, community, health organizations, academic research, and family members can help improve quality of life amongst the elderly.

## Introduction

The numbers of elderly people age 60 years and older are increasing rapidly around the world. It is expected to increase from an estimated 900 million in 2015 to nearly 2 billion in 2050. Most of aging population is found in developing countries ^1^. Thailand is one such developing countries; and the number of elderly people is expected to rise from 13.20% of the total population in 2010, to 32.10% in 2040 ^2^. Phayao province is located in the northern part of Thailand. Phayao Provincial Health office ^3^ reported that the number of elderly people increased from 7.4% in 1992, to 18% in 2017. Many within this population are providing care for their children and grandchildren infected with HIV/AIDS. HIV/AIDS continues to be a major global public health issue, and it has claimed more than 35 million lives so far. In 2017, 940,000 people died from HIV/AIDS globally. There were approximately 36.9 million people living with HIV/AIDS; and almost 1.8 million people became newly infected worldwide in 2017 ^4^

440,000 HIV/AIDS infected patients have been registered with private hospitals in Thailand over the last thirty years. The majority of these patients are between 25–39 years of age and they were unemployed. When classifying the prevalence of HIV/AIDS infection by region, the majority of infected are found in the northern part of Thailand. The prevalence of HIV/AIDS in the northern region is highest amongst commercial (15.10%), and non- professional sex workers (10.20%) ^5^. According to the Phayao Provincial Health office, ^3^ the prevalence of HIV/AIDS infection from 2013 to 2016 was 974.89, 1052.31, 1117.15 and 1161.82 respectively, with incidence rates of 75.75, 72.83, 59.56, and 44.67. Elderly people in Phayao province are currently taking care of family members living with HIV/AIDS. The vast majority of HIV/AIDS’s parents are 50 years and over, and many of them are 60 years and above. The impact of a HIV/AIDS infected person on their caregivers can occur through numerous routes including (1) straining of caregiver and associated opportunity costs, (2) providing financial and material support, (3) raising the survival rate in their grandchildren, (4) suffering from emotional stress, and (5) losing old-age support that the child would have provided ^6^. The study of Sung-Jae Lee, Li Li ^7^ reported that the burden on a HIV/AIDS patient family caregiver in Thailand can be ‘moderate to severe’ or ‘severe’ burden (66.50%).

There are both direct and indirect impacts on the individuals from the spread of HIV on families and communities; for instance, a child becomes an orphan when his/her parent dies from the disease; elders has to take care of HIV/AIDS infected family member; and sometimes elderly people have to take care of the patient’s child in the case of the patient dies or has a physical disability. Attention has mainly focused on the infected group and their children but rarely considers the caregivers who are also greatly impacted ^8, 9^. Knodel, Chanpen ^10^ mentioned that caregivers are most commonly the parents, and it is reported that a parent provides care for almost two thirds of adult who die of HIV/AIDS. Caregivers are not infected with HIV/AIDS, but they have the burden of looking after people living with the disease, often as well as taking care of the patient’s children ^11^.

Therefore, this study focused on quality of life among elderly people affected with family members living with HIV/AIDS. There are 13 districts in Phayao province. Mueang Phayao District is reported to have one of the highest prevalence’s of HIV/AIDs. The majority of elderly people living in this community provide care to HIV/AIDs infected persons, mostly their own children. Elderly people face challenges such as the need to earn extra money to support the family financially, taking care of their grandchildren, dealing with their own physical decline in old age, and chronic illnesses, which in themselves lead to a lower quality of life ^12, 13^. Quality of life can be one of indicators of a healthy life in older age. The World Health Organization (WHO) defines quality of life (QOL) as “an individual’s perception of life in the value system and context of culture in which she or he lives and relation to her or his goals, expectations, concerns and standards”. This study utilized the World Health Organization Quality of Life-Older Adults Module (WHOQOL-OLD) questionnaire to assess quality of life amongst elderly people who are caregivers to individuals infected with HIV/AIDS. The objectives of the study were to assess quality of life amongst this population, and to find factors influencing quality of life amongst elderly people affected by family member living with HIV/AIDS in Phayao province, Thailand.

## Methods

### Study design

This study was a cross-sectional study of older people, aged over 60 years old, who were affected by family members living with HIV/AIDS in Phayao Province, Thailand. The study was performed from January - February 2015. Participants were screened before participating in the study with the inclusion criteria: (a) male or female aged 60 years and older; (b) providing care to family members living with HIV/AIDS disease; and (c) willing to participate in the study. The exclusion criteria included (a) infection with HIV/AIDS, (b) participant having communication problems such as hearing lost; and (c) having a physical disability.

### Sample size and selection

Sample size calculation was done by using G power V.3.1.9.3 ( **α** = 0.05, Effect size = 0.15), Cohen suggest that, if two groups' means don't differ by 0.2 standard deviations or more, the difference is trivial, even if it is statistically significant.

 The required sample size was 138 elderly participants, this was adjusted for a 10% drop-out rate, generating a total sample size of 152 elderly participants. This research was conducted in the areas of high infection rates of HIV/AIDS. Information from the Phayao Provincial Health Office, private clinic data, and health promoting hospital showed that there are 13 sub-districts in the area of Mueang Phayao district reported high rates of HIV/AIDS infection and receiving treatment for HIV/AIDS. A simple random sampling technique was applied to select Ban Tam and Ban Tom sub-districts for this study, from the list of 13 sub-districts. Information of the respondents were taken from health centers data, and they were invited to participate in this study.

### Data collection

Data collectors were trained on how use the data collection tool, and briefed on the study prior to starting the study. The survey technique was face to face interviews. The process took approximately 30 minutes for each participant. The questionnaire included closed-ended questions, and consisted of 2 parts: Part 1. Socio-demographic characteristics questionnaire including gender, age, marital status, education, occupation, income, illness, condition of HIV/AIDS, time taking care of HIV/AIDS infected individuals, social and community activity, and leisure activity in the form of gardening. Part 2. World Health Organization Quality of Life questionnaire-version for older people (WHO QOL-OLD) ^14, 15^. The questionnaire includes 24 items using rating scales and it includes 6 facets: sensory abilities, autonomy, past-present, future activities, social participation, death and dying. Back translation was used to translate questionnaire from English to the Thai language. In terms of validity, the questionnaire was validated by three experts in the field of study. The reliability test of the questionnaire was 0.88. We have assessed the Quality of life into three levels; low, medium and high based on the mean score analyzed from the data. 

### Statistical analysis

Descriptive statistics including frequency, percentage, mean, standard deviation, maximum, and minimum were used to describe general characteristics information of participants. Maximum, minimum, mean and standard deviation were used to display quality of life scores. Correlation coefficient and predictor were used to display relationship of general characteristics information (gender, age, marital status, education, occupation, income, health status, social activity participation, time of taking care HIV/AIDs infected person) and quality of life among elderly people. Stepwise multiple regression was used to assess factors influencing quality of life among the elderly people affected by family member living with HIV/AIDS. Data was analyzed by using SPSS version 20.

### Ethics and consent

All participants received information regarding the research objectives and procedures of the study. Written informed consent was obtained from all participants. All information of participants was kept confidential. The study was approved by Ethics Committee from The College of Public Health Sciences, Chulalongkorn University (case No 193/2558). 

## Results

The majority of participants were female (63.80%), aged between 60–69 years old (mean = 67.20, SD = 52), and married (63.80%). Most of them obtained education at primary school (78.90%). More than half were still working (67.76%), with an average monthly income less than 100 US dollars per month (99.30%). When classified by income, the majority had a sufficient income (67.11%). Most of the participants were free from chronic illness (61.84%), of those who did (38.16%), hypertension (20.68%) and diabetes (23.68%) were the most common. All had suffered from the chronic illness for over a year. More than half of HIV/AIDS infected persons had no disease symptoms 64 (42%). Most of the elderly participants 80 (52%) have been providing care to HIV/AIDS infected family members for 5 years, and 72 (47%) elderly people have been providing care for more than 5 years. Regarding social activity participation, 141 (92%) of the elderly participants have joined community activities and been actively involved in these social gatherings ( Table-1).

**Table 1.** Socio - demographic characteristics of respondents.

Socio-demographic variables	n	%
Gender Male Female	55 97	36.1 63.8
Age (Mean= 67.20, SD= 52, Min 60, Max=75) 60–69 >70	100 52	65.8 34.2
Marital Status Married Widower/Divorce	97 55	63.8 36.2
Education Lower than Primary School Primary School or Higher than primary school	32 120	21.1 78.9
Working Not working Working	52 100	32.2 67.7
Income, US per month (Mean= 1105.92, SD= 670.79, Min 600, Max=5,000) ≤ 100 ≥100	151 1	99.3 00.7
Sufficient Income Yes No	102 50	67.1 32.8
Present illness NO Yes - Hypertension - Diabetes Musculoskeletal disease	94 58 36 19 3	61.8 38.1 23.6 12.5 1.9
Period of time of illness Yes, More than 1 year Yes, Less than 1 year	53 5	34.8 3.2
Number of HIV/AIDS patients in the house 1 More than 1 persons	134 18	88.1 11.8
Currently Number of HIV/AIDS Infected Persons Yes No	83 69	54.6 45.4
Health status of HIV/AIDS Infected Persons Passed away No disease symptoms With disease symptoms	69 64 19	45.3 42.1 12.5
Time taking care of HIV/AIDS patients (Mean=6.61,SD=4.96,Min 1year, Max=25year) Less than 5 years More than 5 years	80 72	52.6 47.8
Participate in any social activities in the community Yes No	141 11	92.7 7.2
Leisure Activity by Gardening Yes No	116 36	76.3 23.9

### Quality of life among participants (QOL)

The results showed that quality of life scores for the elderly participants were either fair (Score of 56-88 score), which the majority of the sample reported (134 participants, 88.20%), or low (Score of 24-55) reported by 18 participants (11.8%). The reported QOL ranged from 44 to 87 ( *x̄*= 73.32, *S.D* = 10.76). When classified into each facet, it showed sensory ability (SAB) score Min= 5, Max = 17 ( *x̄*= 10.63 *S.D* = 2.21), autonomy (AUT) score Min= 5, Max = 17 ( *x̄* = 12.27 *S.D* = 2.33), past, present, future activity (PPF) score Min= 7, Max = 8 *(x̄*= 12.99 *S.D* = 2.50), social participation (SOP) score Min= 8, Max = 18 *(x̄* = 12.27 *S.D* = 2.33), death and dying (DAD) score Min= 4, Max = 17 *(x̄*= 10.61 *S.D* = 2.83), intimacy (INT) score Min= 8, Max = 20 *(x̄*= 13.48 *S.D* = 2.71) ( Table -2 and Table 3).

**Table 2.** Level of quality of life among elderly people (n = 152).

Quality of life Level	n	%
Low level (24–55 scores)	18	11.80
Fair level (56–88 scores)	134	88.20
High level (89–120 scores)	-	-

**Table 3.** Mean and standard deviation of quality of life among elderly people (n = 152).

Outcome Variable	*(n=152)*			
	Mean	SD	Min	Max
Total Quality of life Sensory Ability (SAB) Autonomy (AUT) Past, Present, Future Activity (PPF) Social Participation (SOP) Death and Dying (DAD) Intimacy (INT)	73.32 10.63 12.27 12.99 13.32 10.61 13.48	10.76 2.21 2.33 2.50 2.28 2.83 2.71	44 5 5 7 8 4 8	87 17 17 18 18 17 20

The relationship between predictors regarding the analysis of the linear relationship (Milticolinearity) found each predicted variable had correlation less than 0.08 ( Table 4).

**Table 4.** Correlation coefficient and relationship between predictors shown by correlation matrix.

Variables	1 Gender	2 Age	3 Marital Status	4 Education	5 Occupation	6 Income	7 Health Status	8 Community participation	9 Time providing care	10 Quality of Life among Elderly
1. Gender	1.000									
2. Age	-0.121	1.000								
3. Marital Status	0.015	-0.023	1.000							
4. Education	-0.129	0.078	.0.043	1.000						
5. Occupation	0.026	-0.617 **	0.106	-0.086	1.000					
6. Income	0.061	-0.059	0.015	-0.019	0.057	1.000				
7. Health Status	0.206 **	0.052	0.071	-0.067	-0.065	0.040	1.000			
8. Community Participation	-0.105	0.094	-0.052	0.066	-0.023	-0.087	-0.090	1.000		
9. Time providing care	-0.108	0.177 **	-0.121	0.071	-0.077	-0.149	-0.161	0.011	1.000	
10. Quality of Life among elderly	-0.088	-0.036	0.019	0.042	0.028	0.068	-0.131 *	-0.015	-0.228 **	1.000

* *p* < .05 ** *p* < .01

Stepwise multiple regression analysis with variables including age, marital status, occupation, income, health status, social activity participation and time of providing care is presented in ( Table-5). Time providing care and health status had a statistically significant relationship with quality of life among the elderly with a p-value of 0.05 ( *F = 6.567, p < .01*), and power of prediction of 8.10 ( *R = .285, R Square = .081*).

**Table 5.** Multiple regression prediction of time providing care and health status and quality of life among elderly people.

Source Variance	*df*	*SS*	*MS*	*F*	*p*
**Model 1**					
Regression	1	909.853	909.853	8.230	<.01
Residual	150	16583.700	110.558		
Total	151	17493.553			
**Model 2**					
Regression	2	1417.046	708.523	6.567	<.01
Residual	149	16076.506	107.896		
Total	151	17493.553			

Remarks Model 1 R = .228, R2 Square = .052, S.E = 10.514, n =152          Model 2 R = .285, R2 Square = .081, S.E = 10.387, n =152

Constant and regression coefficients analysis with health benefits of exercise and Quality of life is presented in ( Table-6).

**Table 6.** Constant and regression coefficients of health benefits of exercise and Quality of Life among the elderly people.

Variables	*b*	*SE.*	*Beta*	*t*	*p*
**Model 1**					
Constant	80.550	2.658	-	30.308	<.001
**Time of taking** **care**	-4.900	1.708	-0.228	-2.869	<.01
**Model 2**					
Constant	82.903	2.841	-	29.182	<.001
**Time of taking** **care**	-5.496	1.710	-0.256	-3.215	<.01
Health status	-3.798	1.752	-0.173	-2.168	<.05

## Discussion

This study focused on analyzing variables influencing quality of life among elderly people affected by family member living with HIV/AIDS. Variables including time taking care of the infected individual and health status are key variables influencing quality of life among elderly people within this study. According to previous research ^16^ general characteristics including age, marital status, education, income, and social activity participation have a significant relationship with quality of life in elderly people; however, this study focused on elderly caregivers to HIV/AIDS patients, which may influence the results. It can be concluded that elderly people who have been proving care for family member living with HIV/AIDS disease for a long time, may have a lower quality of life. Knodel ^17^ and Pungchompoo, Pothiban ^9^ reported that as age declines, elderly people have an increased risk of high blood pressure, diabetes, and many health other complications leading to serious illness. Furthermore, they will experience physical decline and cognitive impairment.

Caregiving to HIV/AIDS is a very challenging task; however, when they have to take care of a person with serious illness for such a long time, they may experience fatigue and exhaustion while giving care. Elderly people in this study have to provide emotional and physical support to their family member infected with HIV/AIDS, and the children of infected person; they have been caring for the HIV/AIDS patients for 1–5 years. As a result, they have no time to take care of themselves, and this can exacerbate their own health issues, particularly in those with chronic illness. This is consistent with many previous research studies that indicate elderly caregiver may experience adverse health effects including muscle strain, fatigue, exhaustion, high blood pressure, and/or arthritis when providing extreme day to day care ^9, 10, 18, 19^. This in turn leads to a lower quality of life ^8^. In addition, they also have to earn extra money to support family members which increases the burden on them. Similar to previous studies, we found that the burden of care increases when they have to take care of an ill person for a long time, and they are likely to have a reduced quality of life due to being responsible for providing financial support to the family, and take care of their HIV/AIDS infected family members ^20, 21^

Health status is also one of the predictor’s of quality of life in elderly people. It is found to have a statistically significant relationship with quality of life. It can be concluded that as age increases, their physical ability declines, accompanied by an increased risk of chronic diseases such as hypertension, diabetes and high blood pressure that may lead to a lower quality of life among elderly people ^3, 13^. Previous study found being ill has a significant relationship with quality of life among the elderly (p=0.01) ^16^ which is similar to this study. The study of Nobrega, Jaluul ^22^ found that the QOL among elderly patients who suffered from chronic diseases can be affected by multi-morbidity in the physical domain and probably also in the psychological domain. This study was conducted in selected part of Thailand which cannot be generalized.

## Conclusion

In conclusion, in order to improve quality of life among elderly caregivers to family member living with HIV/AIDS, the time taking care of HIV/AIDS patients, and health status should be focused on. Help and support from the government, community, health organizations, academic research, and family members can help improve quality of life amongst the elderly. In addition heath promoting hospitals and local government should have a home visit program regularly to ensure their needs are met.

## Data availability

### Underlying data

Open Science Framework: Factors influencing quality of life (QOL) amongst elderly caregivers of people living with HIV/AIDS in Phayao province, Thailand: a cross-sectional study, https://doi.org/10.17605/OSF.IO/N7BEK ^23^

Data are available under the terms of the Creative Commons Zero "No rights reserved" data waiver (CC0 1.0 Public domain dedication).
