# Supplementary material for: Factors influencing quality of life (QOL) amongst elderly caregivers of people living with HIV/AIDS in Phayao province, Thailand: a cross-sectional study
Source: F1000Res. 2019 Jan 9;8:39. [Version 1] doi: 10.12688/f1000research.16892.1 (PMC6456834; doi:10.12688/f1000research.16892.1)
Supplement: Peer review history

# Peer review: Reviewer response for version 1

**DOI:** 10.5256/f1000research.18468.r43904

**Published:** 2019-04-05

**Authors:** Umer Farooq

**Affiliations:** 1 Department of Community Medicine, Ayub Medical College, Abbottabad, Pakistan

This is a very useful article and will have policy implications especially in countries with medium to high HIV/AIDS prevalence. The results advocate the importance of the need of support from social agencies and development sectors for the patients of HIV/AIDS to improve their quality of life. I believe the results of their article can be used as a guiding principal.

 The title needs to be rephrased. You may avoid words such as "factors influencing" and "a cross sectional study" – there is no need to put in abbreviations in the title. I believe an appropriate title would be: “Quality of life among elderly caregivers of people living with HIV/AIDS in Phayao Province, Thailand".

 The article is otherwise well written and the study design and the statistical methods employed are correct.

I have read this submission. I believe that I have an appropriate level of expertise to confirm that it is of an acceptable scientific standard.

# Peer review: Reviewer response for version 1

**DOI:** 10.5256/f1000research.18468.r42860

**Published:** 2019-03-22

**Authors:** Faisal Abbas

**Affiliations:** 1 Courant Research Center, “Poverty, Equity and Growth in Developing and Transition Countries”, Department of Economics, George August University, Göttingen, Germany

There are many impacts on quality of life among elderly people living with HIV patients. This study aimed to assess factors influencing quality of life among elderly people living with HIV/AIDS in a northern province of Thailand. This cross-sectional study was conducted in Phayao province, Thailand using systematic sampling technique selecting 152 elderly participants aged 60 years and older with a family member living with HIV/AIDS were recruited to the study. The results of the study showed the mean age of elderly participants was 67.20 + 52 years, most of which were female (97 persons, 63.8%). The mean time taking care of HIV/AIDS patients was 6.61 + 4.96 years. In term of health status among the elderly participants, the majority did not have chronic diseases (61.4%), amongst those with chronic diseases (38.6%), hypertension and diabetes were the most common. The average quality of life score was at a fair level. The time taking care of HIV/AIDS patients and health status were significant predictors of quality of life among participants 8.1 % ( *R ^2^*=.081; *p *< .05). In order to improve quality of life among elderly caregivers to family member living with HIV/AIDS, time taking care of HIV/AIDS patients and health status should be focused on, amongst other factors. Help and support from the government, community, health organizations, academic research, and family members can help improve quality of life amongst the elderly.

 This paper is interesting and written nicely. Data analyses is much improved and according to the objectives of the study. Language is also acceptable and the manuscript in my opinion need no further revision.Therefore, I would like to confirm that the manuscript is acceptable in its current form for indexing in the journal.

I have read this submission. I believe that I have an appropriate level of expertise to confirm that it is of an acceptable scientific standard.
